# Genome-wide comparison deciphers lifestyle adaptation and glass biodeterioration property of *Curvularia eragrostidis* C52

**DOI:** 10.1038/s41598-022-15334-z

**Published:** 2022-07-06

**Authors:** Ngoc Tung Quach, Cao Cuong Ngo, Thu Hoai Nguyen, Phi Long Nguyen, Thi Hanh Nguyen Vu, Thi Hoai Trinh Phan, Quang Huy Nguyen, Thanh Thi Minh Le, Hoang Ha Chu, Quyet-Tien Phi

**Affiliations:** 1 Institute of Biotechnology, Vietnam Academy of Science and Technology, Hanoi, 100000 Vietnam; 2 Graduate University of Science and Technology, Vietnam Academy of Science and Technology, Hanoi, 10000 Vietnam; 3 Vietnam-Russia Tropical Centre, Hanoi, 100000 Vietnam; 4 Department of Marine Biotechnology, Nhatrang Institute of Technology Research and Application, Vietnam Academy of Science and Technology, Nha Trang, 650000 Vietnam; 5 LMI DRISA, Department of Life Sciences, University of Science and Technology of Hanoi, Vietnam Academy of Science and Technology, Hanoi, 100000 Vietnam

**Keywords:** Biochemistry, Genetics, Microbiology

## Abstract

Glass biodeterioration by fungi has caused irreversible damage to valuable glass materials such as cultural heritages and optical devices. To date, knowledge about metabolic potential and genomic profile of biodeteriorative fungi is still scarce. Here, we report for the first time the whole genome sequence of *Curvularia eragrostidis* C52 that strongly degraded silica-based glasses coated with fluorine and hafnium, as expressed by the hyphal surface coverage of 46.16 ± 3.3% and reduced light transmission of 50.93 ± 1.45%. The genome of *C. eragrostidis* C52 is 36.9 Mb long with a GC content of 52.1% and contains 14,913 protein-coding genes, which is the largest genome ever recorded in the genus *Curvularia*. Phylogenomic analysis revealed *C. eragrostidis* C52 formed a distinct cluster with *Curvularia* sp. IFB-Z10 and was not evolved from compared genomes. Genome-wide comparison showed that strain C52 harbored significantly higher proportion of proteins involved in carbohydrate-active enzymes, peptidases, secreted proteins, and transcriptional factors, which may be potentially attributed to a lifestyle adaptation. Furthermore, 72 genes involved in the biosynthesis of 6 different organic acids were identified and expected to be crucial for the fungal survival in the glass environment. To form biofilm against stress, the fungal strain utilized 32 genes responsible for exopolysaccharide production. These findings will foster a better understanding of the biology of *C. eragrostidis* and the mechanisms behind fungal biodeterioration in the future.

## Introduction

Glass, a non-crystalline material, is widely used for practical, technological, and decorative purposes however its originality has relatively been reduced by physicochemical damages or biodeterioration. Once used for long periods of time, the glass is subjected to weathering and drastic changes in appearance, color, and structure^1^. The phenomenon is known as the result of abiotic processes that cause alteration to a glass surface and biotic processes that leach elements from a glass surface through microbial activity^1,2^. With respect to biotic alteration, numerous investigations have been carried out to prove that the damage is also caused by metabolic activities of complex microbial communities including fungi, bacteria, archaea^1,3^. Once microbial community is typically shaped depending on water availability, temperature, and nutrients, biofilm is formed to protect microbial community and mineralize on the surface of glassy material^2^. Microbial cell death can attain physical stress on the glass surface and/or possibly detach, leading to the delamination of all or part of the glass alteration volume. As a result, etching, pitting, leaching, discoloration, and glass degradation are common features of biogenic attack, which are usually observed in medieval stained and optical glasses^3–5^.

Compared to other microorganisms, filamentous fungi play an important role in the biodeterioration of glass. For example, *Aspergillus* sp. is the major fungus causing biodeterioration of historical stained-glass windows in the fifteenth century Cartuja de Miraflores monastery, Spain^1^; *Cladosporium* sp. and *Phoma* sp. are major fungi of Catalonian churches, Spain^3^; *Penicillium* sp. and *Cladosporium* sp. were the two predominant fungal genera isolated from historical glass windows, Portugal^5^. In our previous study, 40 fungal strains classified into 14 genera were recovered from optical observation instruments of museums in Vietnam where *Aspergillus* and *Penicillium* were the most abundant genera^6^. The biodeterioration ability of fungi on glass is identified by a combination of parameters, including organic acids, exopolysaccharides (EPSs), the interaction of carbohydrate-active enzymes, temperature, humidity, and so on. Organic residues present on the glass surface, as dead microbial material, metabolites of autotrophic bacteria, and dust deposits, accompanied by substrates from water vapor in the air initiate the life cycle of filamentous fungi^3^. As a result, biofilm composed of EPSs and specialized substrates is developed to absorb water and increase the chemical destruction of glasses. The surrounding environments are then acidified through the production of organic acids leading to pH changes, redox-reaction leaching and chelation of metal oxides such as Na_2_O, K_2_O, CaO, Al_2_O_3_, and B_2_O, present in glass^3^. As a consequence of bio-pitting corrosion and pitting^3,7^, cracks, Na, K, and Ca were proved to be the most leached elements, while an ionic alteration was recorded for Fe and Mn^5^. Another hypothesis is that the leached elements may react with other components yielding high solubility salts that support fungal growth. As for the medieval stained-window glass, moisture and rain remove the soluble salts, causing patinas and crusts, such as sulfates (gypsum and syngenite), calcite, Ca-oxalates^8^. Until now, most studies have only analyzed colonizing fungal communities on glass and the underlying mechanisms behind fungal biodeterioration remain largely unknown.

*Curvularia* is reported to be a species-rich genus consisting of 131 species listed in Index Fungorum (assessed on 4 January 2018)^9^. The genus *Curvularia* acts predominantly as endophytes and pathogens of plants, animals, and humans^10–12^. Of note, *Curvularia* spp. have been recovered from binocular eyepieces and air, indicating the widespread distribution of this genus in the natural environments^6,13^. Identification by morphology is not enough to delimit species within the genus, thus DNA barcodes and whole-genome sequencing are needed to allow accurate identification and comparison. However, there are only 7 genomes available on Genbank (NCBI), among which *Curvularia* genomes associated with plants occupied the majority. In the previous study, the genome of *C. lunata*, an important pathogen of maize, was sequenced to demonstrate the evolutionary relationship with the fungal pathogen *Bipolaris maydis* and provide a better understanding of pathogen development and infection mechanism^14^. In this study, we isolated and evaluated for the first time glass biodeterioration ability of *C. eragrostidis* C52 of which genome was then sequenced by Illumina platform. Genome-wide analysis and metabolic annotation shed light on lifestyle adaptation and the genetic basis involved in organic acid and EPS production of *C. eragrostidis* C52 under its interaction with the glass surface. The findings from this study will enrich the database of *Curvularia* genome and pave a new way for investigating glass biodeterioration and corrosion by fungi.

## Results

### Evaluation of the glass biodeterioration ability and identification of fungal strain C52

**Figure 1:**
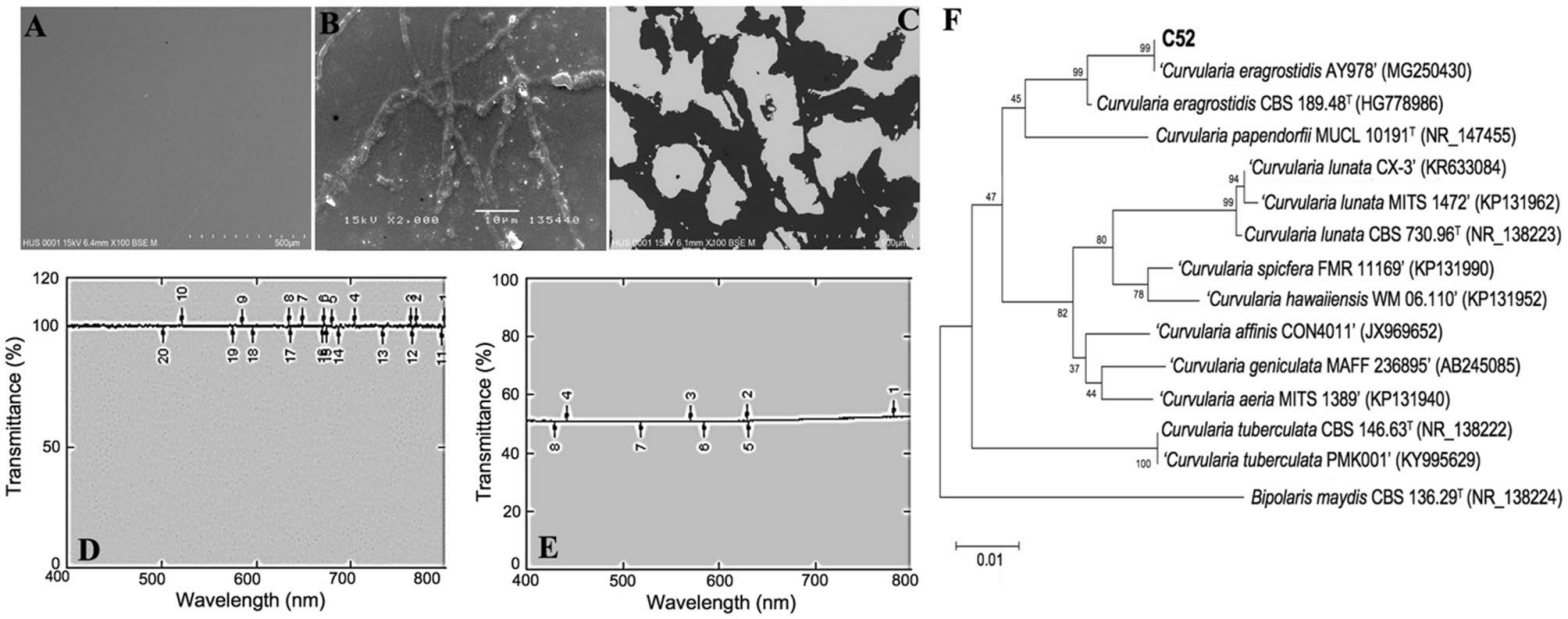
Glass biodeterioration ability and identification of fungal strain C52. Comparison of SEM images of the control (**A**), glass treated by fungal strain C52 before (**B**), and after the cleaning procedure (**C**). Light transmission detected in untreated (**D**) and fungus-treated glasses (**E**). (**F**) Phylogenetic tree based on the ITS gene sequences of *C. eragrostidis* C52 and its related strains.

A total of 12 fungal isolates were isolated from the surface of infected eyepieces of 3 binoculars. Most isolates showed strong growth and acidified the medium to a pH of around 3.0, except for C23, C33, and C34. Glass biodeterioration experiments further revealed that all strains were able to adhere to the glass surface by developing mycelium networks (9.5–46.2%), decrease light transmission through the glass (15.7–50.9%), and produce EPS (0.9–19.0 g/L) (Supplementary Table S1). Among them, fungal isolate C52 caused a remarkable decrease in pH of 2.6, produced the highest EPS concentration (19.0 ± 2.3 g/L), and grew on the glass surface with a hyphal coverage of 46.2 ± 3.3% recorded after 28 days of assessment. SEM analysis showed massive fingerprints on the glass surface by the isolate C52 after the cleaning procedure (Fig. 1A–C). The light transmission of the silica-based glasses infected by the fungal strain C52 was reduced by 50.9 ± 1.4% as compared to the control (Fig. 1D,E). In support of these observations, EDS microanalysis of rough halo caused by hyphae activity indicated a significant increase in potassium, sodium and oxygen, and decrease of fluorine, magnesium, hafnium and especially silicon as compared to the untreated glass. Of note, fluorine and hafnium contents were strongly decreased in fungus-treated glass (Supplementary Table S2). This could be explained by the fact that silica-based glasses used in this study were coated with fluorine and hafnium-based oxide materials to increase surface roughness, the contact angle of water and the sputtering power^15,16^. Taken together, the isolate C52 severely damaged the surface layer of the experimental glasses in short-term incubation.

Morphological observation showed that the colony of strain C52 on a Czapek–Dox agar plate reached a growth rate of 2.3 ± 0.15 cm/day, with abundant aerial mycelium giving a grey cottony appearance with rings on the plate; surface olivaceous grey surface (Supplementary Fig. S1). A reverse plate of strain C52 had olivaceous grey to olivaceous black pigmentation. The hyphae were branches, septate, and thin-walled with the size of 1.5–3 µm in diameter. Conidiophores are represented as singly or in groups, straight or flexuous. The size of cells did not decrease towards the apex and was sometimes branched. The cell walls were thicker than those of vegetative hyphae, mononematous, macronematous, reddish-brown to brown, paler towards apex, up to 700 μm long × 3–6 μm wide. The conidia were ellipsoidal to fusiform, pale brown, apical, and basal cells paler than the middle cells. The conidia varied in size ranging from 11 to 16 μm long × 16–34 μm wide. Furthermore, molecular identification using ITS gene sequencing analysis showed that strain C52 was most related to *C. eragrostidis* AY978 (100% sequence identity, SI), *C. eragrostidis* CBS 189.48^T^ (99.7% SI), and *C. papendorifii* MUCL 10191^T^ (93.6% SI). In the neighbor-joining tree, strain C52 and reference taxa *C. eragrostidis* CBS 189.48 (HG778986) formed a clade with 100% bootstrap support (Fig. 1F). This clade and another reference taxa *C. papendorifii* MUCL 10191 formed a clade with 45% bootstrap support. Combining the morphological and molecular results, the strain C52 was identified to belong to species *Curvularia eragrostidis.*

### Genome sequencing, genome feature, and functional annotation

The genome of *C. eragrostidis* C52 was sequenced using Illumina platform, resulting in 11,419,284 reads containing 1,585,458,996 bases (Supplementary Table S3). The de novo assembly of the *C. eragrostidis* C52 genome yielded approximately 36.9 Mb consisting of 3594 contigs among which the total length of the 200 largest contigs occupied 83.2% of genome size (Table 1). The guanine-cytosine (GC) content of the C52 genome was 52.1%. Based on comparison to BUSCO set of 1312 fungal orthologs, a total of 1233 set was determined in the assembled genome of *C. eragrostidis* C52. Moreover, over 94.0% of BUSCO genes were complete with only 1.0% missing and 5.0% fragmented BUSCOs, suggesting an excellent assembly integrity in the present study. The number of exons and introns were 28,799 and 15,195 genes, respectively with around 2 exons per gene on average. The genome of *C. eragrostidis* C52 was deposited onto the GenBank (NCBI) database under accession number: JAIRCK000000000.

**Table 1:** Comparison of genome features between *C. eragrostidis* C52 and closely related *Curvularia* species.

Feature	C52	P1	30M1	CX-3	UM 226	IFB-Z10
Size (Mb)	36.9	33.0	33.3	33.5	33.4	33.0
G + C content (%)	52.1	50.1	52.1	50.6	50.7	50.5
Number of contigs	3594	574	107	327	374	136
N50	230,720	247,944	1,018,367	788,415	146,099	1,949,676
Protein-coding genes	14,913	10,468	11,004	10,165	10,245	9469
Total exons	28,799	24,851	13,504	24,799	25,233	25,343
Average exon length (bp)	537.02	516.83	540.43	503.07	488.93	505.57
tRNA	174	127	198	124	122	129

*C52, *C. eragrostidis* C52; P1, *C. geniculata* P1; 30M1, *C. kusanoi* 30M1; CX-3, *C. lunata* CX-3; UM 226, *C. papendorfii* UM 226; IFB-Z10, *Curvularia* sp. IFB-Z10.

It was important to determine transposable elements (TEs) since they were reported to be involved in genome size expansion and evolution^17^. In total, TEs were predicted to be approximately 1.1% of the assembled genome in which Class I TEs (retrotransposons) occupied most in the interspersed repeat (Supplementary Table S4). Long interspersed nuclear elements (LINEs) were the most abundant Class I TEs accounting for 0.3%. Tandem repeat sequences represented 0.8% of the assembled genome. *C. eragrostidis* C52 genome also harbored 194 rRNA genes.

The genome was predicted to have 14,913 protein-coding genes with an average sequence length of 1318.5 bp. Among them, 8,622 (57.8%) were successfully assigned to GO terms, 11,883 (79.7%) were similar to the InterPro, 14,207 (95.2%) were mapped to COG, and 10,093 (67.7%) homologs were similar to sequences in Pfam, 7121 (47.8%) were annotated by KEGG pathway (Supplementary Table S4). Among them, the genes in clusters of COG families of *C. eragrostidis* C52 were assigned to 24 functional categories. Genes involved in “function unknown” accounted for the majority (3262 genes), followed by “carbohydrate transport and metabolism” (766 genes), “posttranslational modification, protein turnover, chaperones” (647 genes), and “amino acid transport and metabolism” (631 genes) (Supplementary Fig. S2A). Genome-wide comparison across *Curvularia* genomes revealed that the 4 largest groups found in *C. eragrostidis* C52 were far more than that in *C. kusanoi* 30M1, *C. lunata* CX-3; 6, *C. papendorfii* UM 226; 7, *Curvularia* sp. IFB-Z10, and *C. geniculata* P1 (Supplementary Table S5). This could be due to the discrepancy in the isolation source and life cycle of these fungi. In support of COG analysis, KEGG analysis was conducted to show that “metabolism” was the most abundant group, among which the most representative pathways were “global and overview maps”, “carbohydrate metabolism”, “amino acid metabolism”, and “energy metabolism” (Supplementary Fig. S2B).

### Phylogenomic analysis

**Figure 2:**
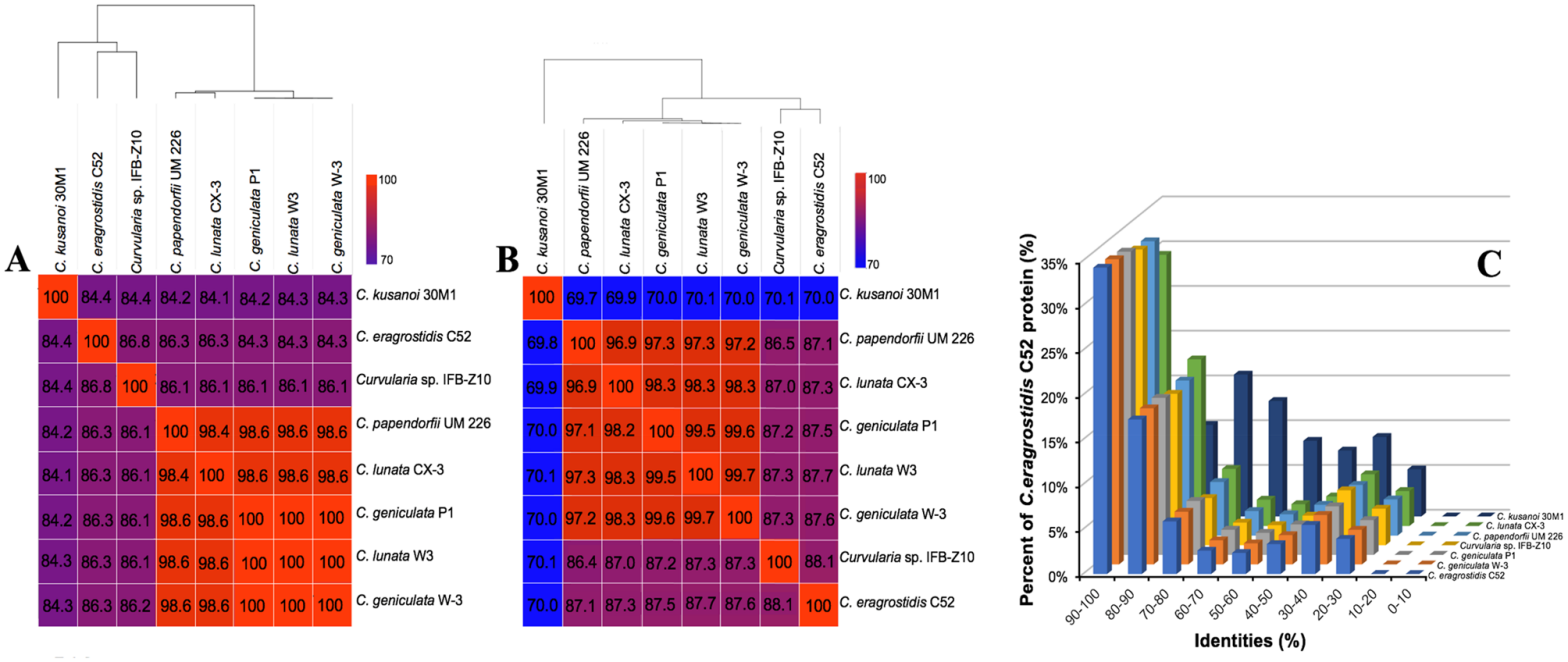
Phylogenomic calculation of the average nucleotide identity (**A**), average amino acid identity (**B**), and amino acid sequence identity (**C**) of *C. eragrostidis* C52 with available genomes within the genus *Curvularia.*

To better estimate the genomic differences between fungal strain C52 and close-related strains, the average nucleotide identity (ANI) which measures the nucleotide-level genomic similarity between two genomes was conducted. The ANI values between members of the genus *Curvularia* varied from 84.1 to 99.9%. The fungal strain C52 exhibited high ANI values ranging from 84.5 to 86.8% as compared to 7 selected genomes, in which the highest ANI value was observed in *Curvularia* sp. IFB-Z10. Phylogenetic analysis revealed the close genetic relatedness between *C. eragrostidis* C52 and *Curvularia* sp. IFB-Z10 (Fig. 2A). Furthermore, we analyzed the average amino acid identity (AAI) that measures the differences among orthologous proteins of 8 *Curvularia* strains. The members of *Curvularia* genera shared at least 70% of their amino acid content (Fig. 2B). The AAI value between *C. eragrostidis* C52 and *Curvularia* sp. IFB-Z10 was by far the highest with 88.1%, indicating that proteins present in the genus *Curvularia* were quite conserved. Amino acid sequence identity analysis showed that approximately 30% *C. eragrostidis* C52 proteins had over 90% of amino acid sequence identity with compared genomes except for *C. kusanoi* 30M1 (Fig. 2C). By contrast, around 12.9% and 8.4% of *C. kusanoi* 30M1 genes had 60–70% and 50–60% of amino acid sequence identity with *C. eragrostidis* C52, respectively.

### Profiling carbohydrate-active enzymes and peptidases

**Figure 3:**
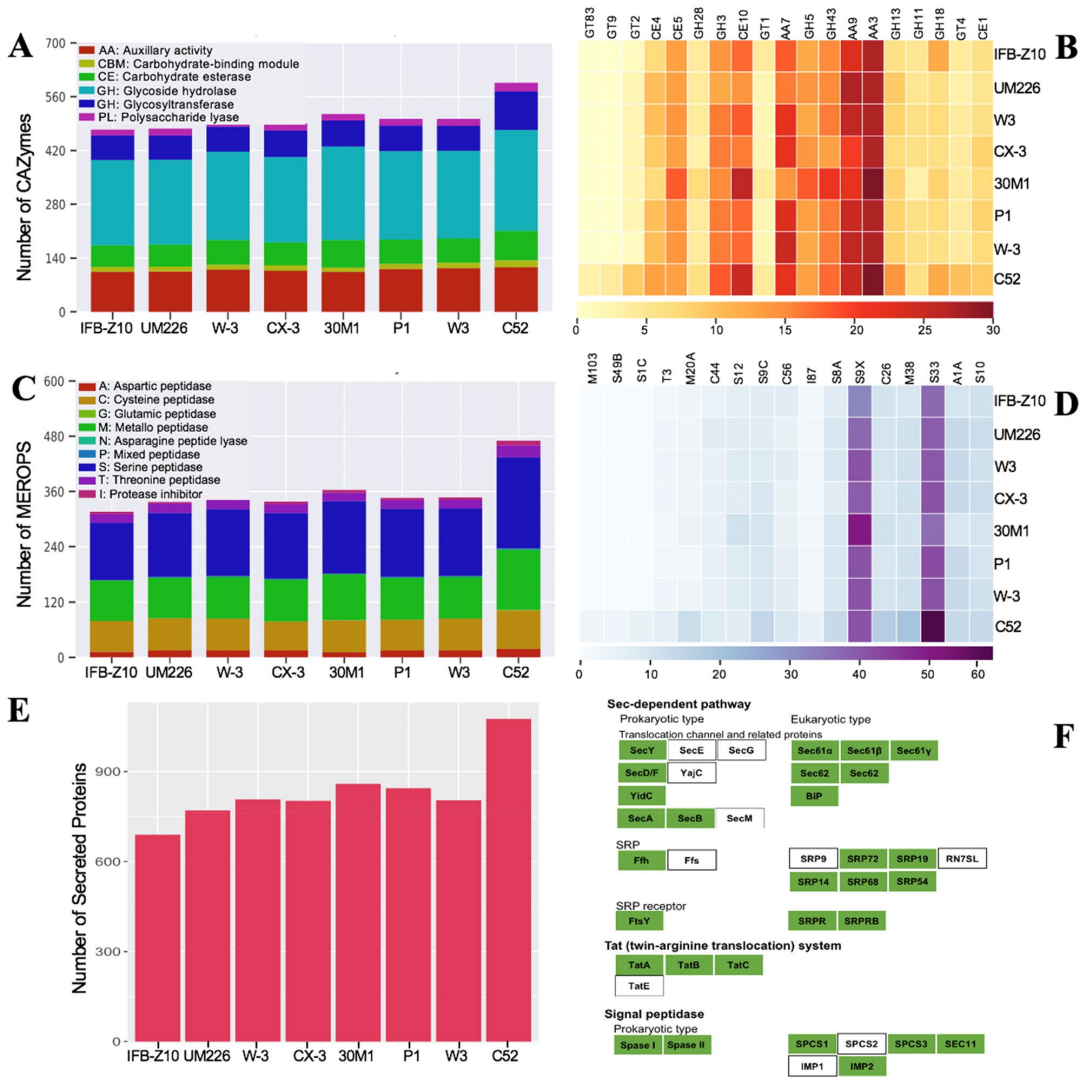
The comparative analysis of CAZymes and proteases from *C. eragrostidis* C52 and 7 *Curvularia* genomes*.* CAZymes distribution (**A**) and heatmap showing the most abundant CAZyme families (**B**) in the proteomes of *Curvularia* species. Distribution of protease families (**C**) and heatmap showing the most abundant protease families found in *Curvularia* genomes (**D**). Secreted proteins (**E**) and protein export predicted by KEGG^19^ (**F**) in *C. eragrostidis* C52. C52, *C. eragrostidis* C52; W-3, *C. geniculata* W_3; 30M1, *C. kusanoi* 30M1; CX-3, *C. lunata* CX-3; W3, *C. lunata* W3; UM226, *C. papendorfii* UM 226; P1, *C. geniculata* P1; IFB-Z10, *Curvularia* sp. IFB-Z10.

In order to identify enzymes responsible for unique lifestyle adaptation, the search for CAZyme domains and distribution across different CAZy families in *C. eragrostidis* C52 was performed through the dbCAN server. The genome of strain C52 revealed the existence of 596 CAZymes with high diversity of families that included 262 glycoside hydrolases (GH), 116 auxiliary activity (AA), 101 glycosyl transferases (GT), 77 carbohydrate esterases (CE), 22 polysaccharide lyases (PL), 18 carbohydrate-binding modules (CBM) (Fig. 3A). As compared to other *Curvularia* genomes such as *C. papendorfii* UM226 (477), *C. lunata* CX-3 (500), *C. kusanoi* 30M1 (515), and *C. geniculata* W3 (502), this was the highest number of CAZymes reported. Given that GH enzymes were crucial for carbohydrate metabolism and organic acid production, the genome of strain C52 contained 75 GH families. GH3 (18 genes) was the most abundant GH family, followed by GH43 (17 genes), and GH16 (14 genes). Additionally, 29 out of 75 GH families comprised only one gene, which was comparable to other *Curvularia* strains. The fungal genome was also found to be rich in AA families that were divided into 8 families of ligninolytic enzymes and 3 families of lytic polysaccharide monooxygenases^18^. In this study, 30 AA3 (glucose-methanol-choline oxidoreductase; alcohol oxidase, aryl-alcohol oxidase/glucose oxidase, cellobiose dehydrogenase, pyranose oxidase), 25 AA9 (lytic polysaccharide monooxygenase), and 22 AA7 (glucooligosaccharide oxidase) were detected as the most abundant CAZyme families (Fig. 3B). Although *C. eragrostidis* C52 had a higher number of GH and AA family members, the profile of GH and AA genes were similar to those in other endophytes including *C. geniculata* W_3, *C. lunata* CX-3, *C. geniculata* P1, *C. lunata* W3 and pathogens including *C. kusanoi* 30M1 and *C. papendorfii* UM 226.

Besides GH and AA, *C. eragrostidis* C52 genome contained 36 GT family members which were involved in the biosynthesis of oligosaccharides, polysaccharides, and glycoconjugates based on catalytic activities on glycosidic linkages^20^. Out of 22 most abundant family members represented by heatmap, GT family occupied 5 members including GT83, GT9, GT2, GT1, and GT4, which was only lesser than GH family (Fig. 3B). Surprisingly, the GT2 glucosyltransferase was 2.5-fold higher than that of other compared strains while GT9 and GT83 had not been observed in other *Curvularia* genomes, which made this the most significant difference observed in this study. A smaller amount of CE, PL, and CBM were identified in all comparative genomes, in which only 4 CE family members occupied predominantly as represented on the heatmap. CE10 was the largest CE family with 26 genes, which was similar to *C. kusanoi* 30M1 but not with others.

To decipher a lifestyle adaptation and glass biodeterioration ability of the strain C52, a BLAST search against MEROPS protease database was carried out for all selected fungi. A total of 470 peptidases were classified into 8 big families, including serine (198 genes), metallo peptidase (130 genes), cysteine peptidase (84 genes), theronine peptidase (26 genes), aspartic peptidase (18 genes), protease inhibitors (10 genes), mixed peptidase (2 genes), glutamic peptidase (1 gene), asparagine peptide lyase (1 gene), accounted for 119 sub-families (Fig. 3C). Comparative genome analysis further showed that *C. eragrostidis* C52 had the highest number of predicted proteases among *Curvularia* species with the number of peptidases ranging from 316 to 363. All studied genomes showed similar proteolytic profiles and only *C. eragrostidis* C52 harbored a gene related to glutamic peptidase and asparagine peptide lyase. Out of 119 protease families, the largest family was prolyl aminopeptidase S33 (59 genes), followed by serine protease S9X (43 genes) (Fig. 3D). The unique families of proteases are expanded in *C. eragrostidis* C52, indicated by the presence of 10 serines (S1C, S11, S13, S24, S41A, S45, S49B, S49C, S73, S85), 14 metallo peptidases (M4, M14D, M15C, M17, M23B, M29, M30, M48B, M50B, M55, M61, M81, M90, M103), and 5 other families (A8, A24A, T01B, N06, C40) that were absent in 7 compared genomes.

### Secreted proteins and protein export

Since secreted proteins were necessary for fungus-host, microbe-microbe and fungus-environment interactions, genomic analysis was implemented to predict secreted proteins in *C. eragrostidis* C52 that contained 1076 sequences (Fig. 3E). Genome-wide comparison showed an expansion of secreted proteins identified in glass-derived fungus C52 as compared to endophytic fungi *C. lunata* CX-3 (803 proteins), *C. geniculata* P1 (845 proteins), *C. lunata* W3 (807 proteins), *C. geniculata* W_3 (804 proteins) or human pathogenic fungi *C. kusanoi* 30M1 (859 proteins), *C. papendorfii* UM 226 (770 proteins). In addition, eukaryotic and prokaryotic pathways of protein export were found in the C52 genome, including Sec-dependent pathway, twin-arginine translocation system, and signal peptidase. Out of 39 genes, 10 genes encoding for SecE, SecG, YajC, SecM, Ffs, SRP9, TatE, RN7SL, SPCS2, and IMP1 were not predicted (Fig. 3F).

### Metabolic pathways involved in the organic acid production of *C. eragrostidis* C52

Since the ability to produce organic acids by harmful fungi contributed to glass biodeterioration, organic acid production in the culture medium and metabolic annotation were studied. Strain C52 acidified the MT1 medium to pH of 2.6 and produced various organic acids including citric acid, fumaric acid, gluconic acid, oxalic acid, succinic acid, itaconic acid, and lactic acid. Among them, succinic acid was accumulated in large quantities (0.25 ± 0.03 g/L), followed by oxalic acid (0.15 ± 0.031 g/L) and citric acid (0.07 ± 0.016 g/L) (Table 2).

**Table 2:** Organic acid production profile determined in *C. eragrostidis* C52.

Organic acid	g/L
Citric acid	0.07 ± 0.016
Fumaric acid	0.032 ± 0.004
Gluconic acid	0.02 ± 0.002
Oxalic acid	0.15 ± 0.031
Succinic acid	0.25 ± 0.03
Itaconic acid	0.01 ± 0.002
Lactic acid	0.013 ± 0.003

**Figure 4:**
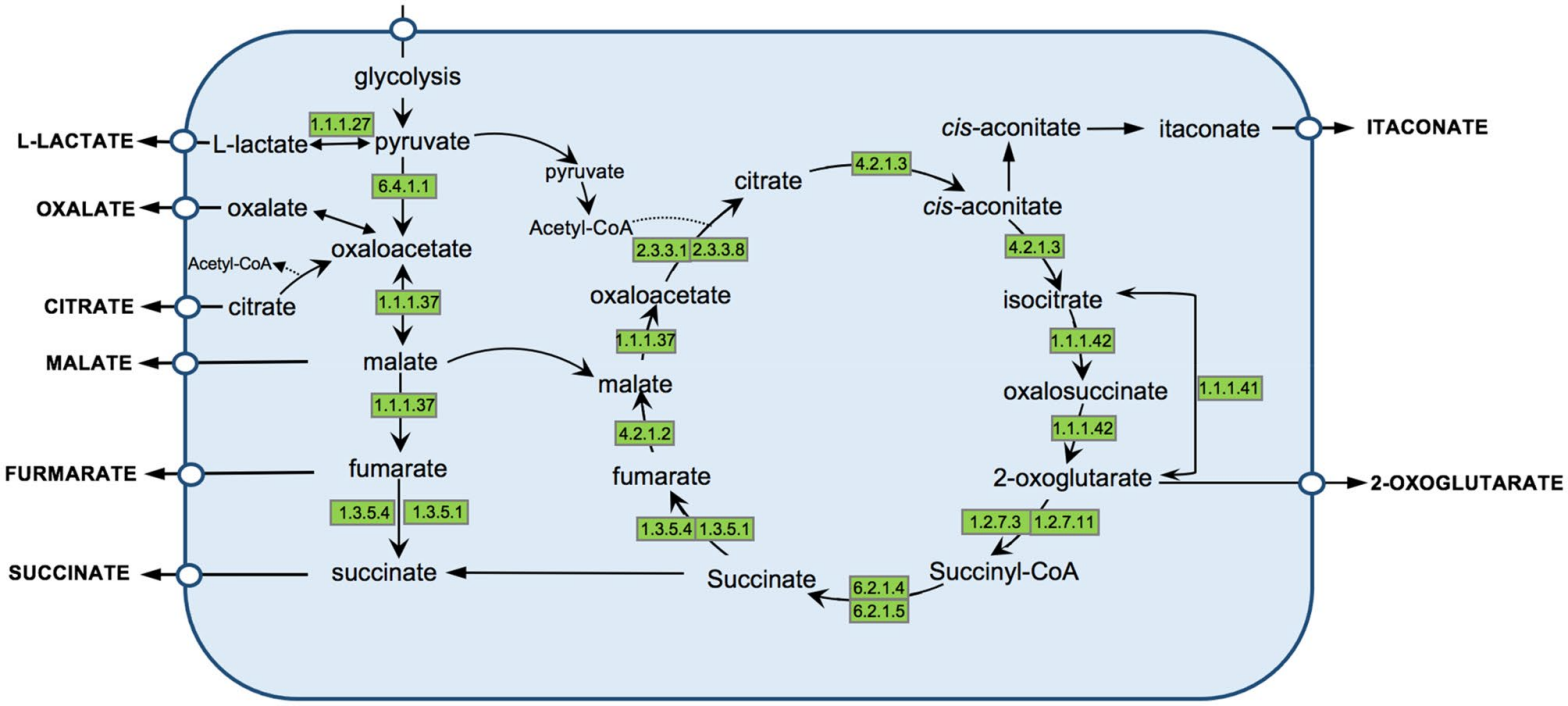
The proposed metabolic pathways involved in organic acid production by *C. eragrostidis* C52.

Metabolic annotation by KEGG revealed 72 genes related to the production of organic acids. From the beginning, carbon and nitrogen sources were extracellularly degraded and fluxed to the glycolysis pathway in order to provide pyruvate. A part of pyruvate was reversely converted to l-lactate through 2 copies of lactate/malate dehydrogenase (*orf_6854, orf_8541*) yielding lactic acid (Fig. 4). Five genes encoding pyruvate carboxylase (*orf_5204, orf_5205, orf_11122, orf_12135, orf_12853*) contribute to oxaloacetate accumulation in the cytoplasm, which were catabolized by malate dehydrogenase (*orf_5034, orf_8145, orf_12974*) to malate and fumarate that are exported outside the membrane. At the end of this metabolic route, the presence of 11 succinate dehydrogenase *sdh* and one l-aspartate oxidase *nadB* might significantly improve succinic acid yield and productivity (Supplementary Table S6). Depending on induction condition, the rest of the pyruvate was converted into acetyl-coenzyme A (acetyl-CoA), which was the substrate of 8 citrate synthases (*orf_3903, orf_9295, orf_11022, orf_14255, orf_12445, orf_12690, orf_1747, orf_1748*). Due to citrate acceleration, citrate was transported to cytoplasm provoking strong oxalic acid production as indicated by phenotypic result above. Parallelly, citrate was commonly catabolized by aconitase (*orf_5714*) and aconitate hydratase (*orf_11512, orf_12715*) to produce *cis*-aconitate following the TCA cycle (Fig. 4). Then, *cis*-aconitate acted as precursor for itaconic acid production. As an intermediate of the TCA cycle, 2-oxoglutarate and succinate were also produced and then exported to the supernatant.

### Exopolysaccharide biosynthesis

To deeply understand biodeteriorative mechanisms of the fungal strains C52, polysaccharide biosynthesis was investigated at both genotypic and phenotypic levels. Increasing the incubation time led to a rise in EPS production. The highest EPS concentration (19 ± 0.2 g/L) was recorded after 7 days of incubation (Supplementary Fig. S3). After 10 days of incubation, a relative decrease was observed (16 ± 1.2 g/L). In support of this result, a total of 32 genes contributing to EPS biosynthesis were identified in the genome of *C. eragrostidis* C52 (Table 3). Given that glucan was an important structural EPS of fungi, the key genes involved in the biosynthesis of glucans were predicted, which included 4 phosphomannomutases/phosphoglucomutases (*orf_2286, orf_9514, orf_11334, orf_14040*) and 2 UDP-glucose-1-phosphate uridylyltransferases (*orf_7127, orf_11944*) genes. One gene encoding β-1,3-glucan synthases (*orf_6760*) and β-glucan biosynthesis-associated proteins (*orf_8141*) was directly involved in β-1,3-glucan and β-1,6-glucan biosynthesis, respectively. The linear glucans were delivered to the outside membrane and then elongated by 2 copies of β-1,3-glucanosyltransferases (*orf_2453, orf_2658*) that belong to the GH72 family. In parallel, the short linear glucans were conjugated to another short 1,3-β-glucan by 5 glycoside hydrolases (*orf_1797, orf_2193, orf_3070, orf_6606, orf_9296*) through a linear β-(1,6)-linkage. Moreover, some genes associated directly and indirectly to β-1,3-glucan and β-1,6-glucan biosynthesis were also predicted (Table 3). Another type of EPS was exopolysaccharide galactosaminogalactan (GAG) that plays an important role in the maintenance of the extracellular matrix of fungal biofilms such as *Aspergillus fumigatus*^21^. However, only deacetylase Agd3 (*orf_4191*) that functioned in the deacetylation of the synthesized GAG polymer was found. Searching on bacteria-type EPS also revealed the presence of 8 genes including 3 copies of *epsA* and 1 copy each of *epsL, epsR, exoA, exoM, exgA,* and *waaL*.

**Table 3:** Genes involved in exopolysaccharide biosynthesis.

Locus tag	Gene name	Predicted function
Orf_2286, Orf_9514, Orf_11334, Orf_14040	*algC*	Phosphomannomutase/phosphoglucomutase
Orf_7127, Orf_11944	*galU*	UDP-glucose-1-phosphate uridylyltransferase
Orf_6760	*fks*	1,3-Beta-glucan synthase
Orf_8141	*bms*	Beta-glucan synthesis-associated protein
Orf_2658	*gas4*	1,3-Beta-glucanosyltransferase
Orf_2453	*gas1*	1,3-Beta-glucanosyltransferase
Orf_2193, Orf_1797	*bgl1-2*	Beta-glucosidase 1A
Orf_9296	*bglE*	Glycoside hydrolase family 3 protein
Orf_6606	*bglF*	Glycosyl hydrolase 3 family protein
Orf_3070	*bglG*	Glycoside hydrolase 3 family protein
Orf_4191	*agd3*	GTPase-activating protein
Orf_9625	*-*	Calnexin
Orf_1356	*cdc10*	GTP-binding protein
Orf_11266	*typA*	GTP-binding protein
Orf_1846	*-*	GTPase-activating protein
Orf_49	*sac7*	Rho-GTPase-activating protein
Orf_10144	*rga1*	Rho-GTPase-activating protein
Orf_3136	*rot1*	ROT1 protein
Orf_10844, Orf_14145, Orf_14645	*epsA*	EPS I polysaccharide outer membrane protein
Orf_13556	*epsL*	PFAM sugar transferase
Orf_11225	*epsR*	Helix-turn-helix protein
Orf_13776	*exoA*	Exodeoxyribonuclease III
Orf_14570	*exoM*	Glycosyltransferase like family 2
Orf_1781	*exgA*	Glucan 1,3-beta-glucosidase
Orf_12624	*waaL*	O-Antigen ligase

## Discussion

Most of the current investigations have been focusing on the medical and agricultural applications of fungi, little is known about glass deterioration of fungi, especially genetic information. With the development of next generation sequencing techniques, genome research of fungi has recently started getting more attention due to the complexity of genomic and physiological characteristics. In this present study, we reported for the first time a genome sequence of *C. eragrostidis* C52 that produced clear damages on silica-based glasses coated with fluorine and hafnium elements, presented as fingerprints. The findings provide a better understanding of genomic features of *C. eragrostidis* C52 in deteriorating optical glass.

The genome assembly revealed that *C. eragrostidis* C52 is the largest sequenced genome within the genus *Curvularia.* At the time of writing, there are only 7 *Curvularia* genomes sequenced and deposited onto GenBank (NCBI). The *C. eragrostidis* C52 genome size of 36.9 Mb is by far larger than other reported sizes ranging from 33 Mb (*C. geniculata* P1) to 35.5 Mb (*C. lunata* W3). At the species level, *C. eragrostidis* C52 is the first sequenced genome. The number of predicted protein-coding genes of *C. eragrostidis* C52 (14,913) was remarkably higher than that of other species such as *C. lunata* W3 (33.5 Mb, 10,165 protein-coding genes), *C. kusanoi* 30M1 (33.3 Mb, 11,004 protein-coding genes), and *Curvularia* sp. IFB-Z10 (33 Mb, 9,469 protein-coding genes). TEs are known as mobile genetic units, which cause mutations, gene expression and chromosomal rearrangement, thereby aiding the populations to adapt successfully to changes in the environment^22,23^. The genome size of plant pathogens such as *Phytophthora infestans,* and *Blumeria grami* f.sp. *hordei* is expanded due to the abundance of TE proliferation that accounted for approximately 29% of the genome^24,25^. In addition, TE repertoires vary not only among genus levels but also in the closely related fungal species. TE was estimated to be 1.1% of the *C. eragrostidis* C52 genome, which was not different from other *Curvularia* genomes. These results suggested that the expansion of protein-coding gene inventory might lead to the larger genome size of *C. eragrostidis* C52, which may be due to the lifestyle and ecological niche. Apart from that, TEs also function as novel promoters interfering in transcription processes that play an important role in fungal development and evolution^26^. Genomic analysis determined 372 genes related to transcription factors (TFs) accounting for approximately 0.03% of the total predicted genes, which is relatively similar to 7 compared genomes. Although the TF profile of C52 was compatible to others, lambda repressors containing helix-turn-helix domain, PAS fold, and bacterial regulatory HTH proteins were around fourfold, sixfold and 18-fold higher, respectively, than those of genes in other compared genomes (Supplementary Fig. S4). Hence, this finding could help explore the evolutionary relationships and lifestyle adaptation of *Curvularia* species upon different ecological niches that remain to be investigated in the future.

*C. eragrostidis* C52 displayed a great expansion of certain gene families potentially involved in lifestyle adaptation and colonization. Given that airborne fungal spores have to germinate and develop into hyphae to colonize on glass surface^6^, *C. eragrostidis* C52 might successively undergo asexual and sexual stages. The sexual stage remains unknown, while the asexual stage of *Curvularia* species is reported to be crucial for causing disease to the hosts such as plant and human. As for the important maize pathogenic fungus *C. lunata* CX-3, the colonization process including the attachment to the plant surface, the germination on the plant surface and the formation of infectious structures, and the penetration and colonization of the host tissue attributed to a high proportion of CAZymes, proteases, and secreted proteins^14^. *C. eragrostidis* C52 was rich of CAZymes responsible for colonization cycle, nutrient acquisition and dispersal that are shaped by utilized substrate, lifestyle, and host preference^27^. A recent study proved that the repertoire of CE1 and CE10 genes was significantly reduced in biotrophic pathogens^28^. As it stands, plant-derived *Curvularia* strains (*C. lunata* CX-3, *C. lunata* W3, *C. geniculata* P1, *C. geniculata* W_3) and human-derived strains (*C. kusanoi* 30M1, *C. papendorfii* UM 226) had an average CE10 of 16 genes. By contrast, a higher number of CE10 (26 genes) was shown in *C. eragrostidis* C52 recovered from glass. It could be found through genome-wide comparison that 23 CAZy family members were missing in 7 compared genomes, but not in *C. eragrostidis* C52. Furthermore, serine protease genes also contribute to adaptive natural selection and genome expansion as proved in the mycoparasitic and nematode-parasitic fungus *Clonostachys rosea*^29^. The ability to degrade lignocellulosic substrates such as dead plant material and cell wall components of *Trichoderma* is subjected to the expansion of proteases and CAZymes^30^. In this study, significantly expanded gene sets of S08A, S09C and S33 serine proteases were observed in C52 genome compared to others. Given that strain C52 is the first fungal genome sequence associated with glass biodeterioration, the remarked question is that whether these protease families are coupled to the glass biodeterioration lifestyle and evolutionary history within *Curvularia* genera.

The EPS production by *C. eragrostidis* C52 also contributed to the biodegradation of glass. As described previously, EPSs such as β-1,3-glucan were required for cell walls and especially biofilm development, resulting in colonization of the fungi on the surface of glass^31^. In *C. eragrostidis* C52 genome, the presence of β-1,3-glucan synthase (*Fks*), β-glucan synthesis-associated protein (*Bms*), Rho-GTPase-activating protein (*sac7*, *rga1, rot1*), glycosyltransferase (*gas1, gas4*), and glycoside hydrolase (*bgl1, bglE, bglF, bglG*) resulted in the production of β-1,3-glucan. By contrast, only one out of 4 genes attributed to exopolysaccharide GAG formation was predicted, and some genes related to bacterial EPSs also were also determined. These results suggested that *C. eragrostidis* C52 might produce different kinds of EPS to survive and thrive on the glass, which corresponds to the high concentration of EPS obtained from the supernatant. Since fungal biodeterioration mechanisms are poorly characterized to date, the involvement of EPSs in fungal biodeterioration will still be an interesting subject for future studies.

Organic acids were shown to be secreted by *C. eragrostidis* C52 at the aspect of phenotypic and genotypic levels. It is believed that fungal colonization on the glass through biofilm formation is linked to EPS production, which subsequently produces various organic acids to dissolve metal oxides such as Na_2_O, K_2_O, CaO, Al_2_O_3_, B_2_O, and ZnO^6,32^. Another nutrient source is the lipid layer that comprises long-chain hydrocarbons, fatty acids, and some alkaline metals such as Li, Na, K, Ca, Ba, Al, Zn, and Pb. Under sufficient conditions, fungal growth results in glass corrosion. *A. niger* was reported to produce oxalic acid, formic acid, tartaric acid, malic acid, and citric acid at every pH value, while organic acid secretion produced by *Penicillium ochrochloron* and *Penicillium oxalicum* was inhibited under acidic pH^33,34^. It is clear that organic acid production is pH- and strain-dependent manners. In this study, when grown on the mineral medium containing 2.5 g/L glucose, *C. eragrostidis* C52 was able to produce 7 different organic acids at acidic pH, which was higher than filamentous fungi reported previously that were found to produce 3–6 different organic acids in rich liquid medium^33,35^. In line with this, low organic acid concentrations were produced by the strain C52, which could be due to low glucose concentration supplemented in the culture medium. The presence of organic acids was in agreement with metabolic pathways annotated in the genome of strain C52, except for gluconic acid. A recent study demonstrated that organic acids were excreted fully protonated or as anions accompanied by protons expelled via the plasma membrane H^+^-ATPase, which served important purposes such as charge balance, energy spilling, chelation of trace elements, and nutrient uptake. In some fungi like *Schizosaccharomyces pombe, Hansenula anomala, Penicillium ochrochloron*^34,36,37^, the reuptake of organic acids was observed. Since iron is required for virtually all biological systems, in *A. niger*, citric acid was demonstrated to function as iron siderophore to increase the bioavailability of iron represented as Fe(III) citrate^38^. Moreover, arbuscular mycorrhizal fungi such as *Rhizophagus irregularis* secretes a significant amount of organic acids (acetic, butyric, lactic, citric gluconic, malic, and oxalic acids) to sequester low-accessible phosphorus from Fe oxides^39^. Combining our results, it seems that the production of organic acids is such a critical mechanism to help fungi survive under metal-rich environments.

To date, a number of fungi related to glass decay have been isolated and identified but at the time of writing, the underlying mechanisms and genome sequence of such fungal strains have not been revealed. In the present study, the genome of biodeteriorative fungus *C. eragrostidis* C52 was sequenced to strengthen the *Curvularia* genome database and to provide a better understanding of factors involved in biodeterioration mechanisms. Although the genomic evolution within *Curvularia* genera upon different ecological niches has been mentioned^14^, it is necessary to sequence whole genomes of more species to illustrate this hypothesis more accurately.

## Methods

### Isolation of C52 and evaluation of its glass biodeterioration

Three binoculars highly contaminated by fungi (model 6nu5 8 × 30 M) were collected from Bien Hoa city, Vietnam during 2018–2019, which were then placed in sterile plastic bags and transported to the laboratory for isolation. Fungal strains were derived from the surface of eyepieces comprising multi-layer anti-reflective coatings made from essential elements such as fluorine and hafnium as described by Ngo et al.^6^. In brief, sterile cotton swabs were used to wipe on the surface of eyepieces, then transferred to a sterile tube containing 1 mL of 0.05% Tween 80, and homogenized by shaking at 200 rpm for 30 min. About 100 μL of aliquots were spread on a Czapek–Dox agar medium for 4–5 days at 30 °C. Hyphae tip of the grown fungal colony was cut by a syringe needle (29 gauge) under a stereo microscope and then transferred it onto new Czapek–Dox agar plates. The pure isolates were maintained on a Czapek–Dox agar medium at 4 °C or silicone beads at − 20 °C. Evaluation of the glass biodeterioration by fungal isolates was carried out following the guideline of the ISO 9022-11:2015 document (https://www.iso.org/standard/67535.html, Accessed April, 2015). In brief, fungal spores yielded on potato dextrose agar (PDA) medium were resuspended in a mineral-salt medium containing 0.05% Tween80^6^ to attain approximately 10^6^ spores/mL. After that, the spore solution was spread equally on the surface of silica-based glasses (10 × 20 cm) that had fluorine and hafnium coatings. The control samples were prepared in the same way, in which fungal spores were not added to the glass. These experimental glasses were kept at 30 °C with relative humidity of 90% for 28 days. The hyphal surface coverage was analyzed using a digital camera via OPTIKA Vision Pro and ImageJ v.1.51 softwares^3,8^. The colonization of fungal strain on glass samples was visualized under a JEOL 5410 scanning electron microscope (SEM) (Japan). The light transmittance through glass samples was measured by a spectrophotometer UV-2550 at wavelengths of visible light range from 400 to 800 nm^6^. Elemental compositions on the glass surface of untreated and fungus-treated glasses were quantified by the Oxford instruments Energy dispersive X-ray spectroscopy (EDS) microanalysis system (Oxford Instruments, Buckinghamshire, UK) carried out alongside scanning electron microscopy (SEM) (Hitachi, Tokyo, Japan) with default parameters at 15 kV accelerating voltage^40^. The weight and atomic percentages of elements were calculated as described previously^41^.

### Identification of harmful fungi

The strain C52 was incubated on the Czapek–Dox agar medium at 30 °C for 4–5 days to identify the morphological characteristics as described previously. Genomic DNA of fungal strain C52 was extracted using the microwave method as described previously^42^. PCR was then performed to amplify the internal transcribed spacer (ITS) region sequences by using primer pairs ITS1F (5′-CTT GGT CAT TTA GAG GAA GTA A-3′) and ITS4 (5′-TCC TCC GCT TAT TGA TAT GC-3′). Purification and sequencing of PCR product were done by FIRST BASE Laboratories Sdn. Bhd. (Malaysia). The resulting sequence was analyzed by using BioEdit v7.2.5 and compared with those available in GenBank via BLASTn search on GenBank (http://www.ncbi.nlm.nih.gov/). Phylogenetic analyses were conducted using the neighbor joining (NJ) methods in MEGA v7.0 and the bootstrap was 1000 replications to assess the reliability level of the nodes tree^43^. The type strain *Bipolaris maydis* CBS 136.29 (NR_138224) was used as the outgroup branch.

### EPS production

EPS production was determined as g/L according to the procedure described by Jaroszuk-Ściseł et al. (2020)^44^. Strain C52 was grown on a Czapek–Dox broth agar medium for 72 h at 30 °C. The culture was used as inoculum in 100 mL of Czapek–Dox medium containing 5 g/L glucose. At intervals, a sample was taken, and EPS was then precipitated by the addition of an equal volume of chilled ethanol to the cell-free supernatant. After keeping at 4 °C for 24 h, the precipitated EPS was washed with chilled ethanol, freeze-dried, and weighted to determine the raw EPS concentration.

### Organic acid production analysis

*C. eragrostidis* C52 was cultivated on a Czapek–Dox medium at 30 °C for 3 days as the seed culture. Assessment of the ability to produce organic acids of the strain C52 was carried out in 500 mL conical flasks containing 200 mL MT1 medium (glucose 2.5 g, (NH_4_)_2_SO_4_ 0.75 g, MgSO_4_·7H_2_O 1.0 g, NaCl 1.0 g, CaCl_2_·2H_2_O 0.1 g, 0.5 ml trace element (1.3 g CuSO_4_·5H_2_O, 6.9 g FeSO_4_·7H_2_O, 3.5 g MnCl_2_·4H_2_O, 7.2 g ZnSO_4_·7H_2_O, 0.5 g NiCl_2_·6H_2_O), pH 7.2, in a liter) at 30 °C for 6 days with an inoculation rate of 2% (v/v). Cell-free supernatant was harvested by centrifugation and then passed through ultra-filtration using Vivaspin 5kD (VWR, Strasbourg, France) tubes to eliminate proteins. Organic acids in the solution were determined using gas chromatography-mass spectrometry (GC–MS) with some modifications^45^. Briefly, 2.5 M NaOH was added to alkalize the solution, and 200 μL of the alkalized solution was then derivatized by the addition of methylchloroformate which was extracted with dichloromethane. About 2 μL of the extract were injected in the split injector port of Agilent 5973 N system, equipped with an Omegawax (Supelco, Bellefont, PA, USA), 30 m × 250 μm i.d. × 0.25 μm thick films. Before injection, the oven temperature was programmed (40 °C for 3 min, then 8–230 °C at 10 °C/min) and was isothermally held for 15 min. Compounds were determined after comparing with available data in the NIST library and using the standard organic acids proposed.

### DNA extraction, genome sequencing, and de novo aseembly

The mycelia of *C. eragrostidis* C52 were harvested from liquid cultures grown in a Czapek–Dox medium at 30 °C for 3 days for DNA extraction. Mycelia were harvested and ground into a fine powder by using liquid nitrogen. The genomic DNA was extracted from the resulting powder using DNeasy Plant Minikit (Qiagen, Hilden, Germany) according to the manufacturer’s instructions. DNA quantity and purity were assessed using a Nanodrop UV–Vis spectrophotometer (Thermo Fisher Scientific, USA) and a Qubit 2.0 fluorometer (Life Technologies, United States). The whole genome of *C. eragrostidis* C52 was de novo sequenced using Illumina MiSeq platform. Approximately, 11.4 million paired-end raw reads were generated and then quality was checked by FastQC. Low-quality reads (< Q15) and adapters were removed using Fastp v0.20.0. De novo assembly of Illumina reads data was performed using SPAdes v3.15.1 (https://github.com/ablab/spades) and MegaHit v1.2.9 (https://github.com/voutcn/megahit) with default parameters. The completeness of the fungal genome was analyzed by BUSCO v3^46^.

### Gene prediction and annotation

Genome annotation of *C. eragrostidis* C52 was performed using Funannotate v1.8.1 (https://github.com/nextgenusfs/funannotate/tree/v1.8.1) which employs GeneMark-ES v4.48_3.60_lic^47^, Augustus v3.3.2^48^, SNAP v2006-07-28^49^, and GlimmerHMM v3.0.4^50^. Ribosomal RNA and tRNA genes were predicted using RNAmmer v1.2^51^ and tRNAscan-SE v2.0.5^52^. Noncoding RNAs (ncRNAs) in the genome of fungal strain C52 were predicted using Infernal v1.1.2^53^. The repetitive elements in the C52 genome were identified by RepeatModeler v2.0.1^54^. Pseudogene was determined using PseudoPipe^55^. All desirable open reading frames (ORFs) were blasted against the NCBI curated refseq_protein database (reference proteins) by Blastp^56^. Gene Ontology (GO) and Cluster of Orthologous Groups (COG) were annotated based on EggNOG-mapper v1.0.3^57^. KEGG Automatic Annotation Server (KAAS) was performed by an assignment method of ‘bi-directional best hit’ against data of any species on the KEGG database to identify functional protein sequences following KEGG Orthology^19,58^. Default parameters were applied for all software tools.

### Comparative genome and phylogenomic analysis

Seven *Curvularia* genomes available on GenBank (NCBI) divided into 3 groups including plant-derived strains (*C. lunata* CX-3, *C. lunata* W3, *C. geniculata* P1, *C. geniculata* W_3), human-derived strains (*C. kusanoi* 30M1, *C. papendorfii* UM 226), and fish-derived strain (*Curvularia* sp. IFB-Z10) were used for comparative genome analysis. Orthologous protein domains between *C. eragrostidis* C52 and selected fungal genomes were determined using OrthoMCL (version 2.0.9)^59^. Furthermore, the matrix of ANI and AAI was carried out using CompareM v0.1.2 (https://github.com/dparks1134/CompareM) and PYANI v0.1.2 (https://github.com/widdowquinn/pyani/releases/tag/v0.1.2), respectively to identify the relationship within *Curvularia* species.

### Protein family classifications

Identification of genes related to Carbohydrate-Active enZYme (CAZyme) families including glycoside hydrolase (GH), carbohydrate-binding module (CBM), glycosyl transferase (GT), polysaccharide lyase (PL), carbohydrate esterase (CE), and auxiliary activity (AA) in the genome of C52 and 7 other selected genomes was carried out using the dbCAN meta server (http://bcb.unl.edu/dbCAN2/) including the dbCAN CAZyme domain (HMMER search), short conserved motifs (Hotpep search), and CAZy databases (DIAMOND search)^60^. All positive hits were manually checked for final validation. The MEROPS peptidase database^61^ was applied to classify enzyme classes including aspartic, cysteine, glutamic, serine, metallo, threonine, asparagine, mixed peptidase, and protease inhibitor. Protein sequences with positive hits were checked again with a BLAST search (e-value cut-off = 1e−04) against the NCBI NR protein database. Apart from that, secreted proteins were identified based on the combination of SignalP v4.1^62^, TargetP v2.0 server (https://services.healthtech.dtu.dk/service.php?TargetP-2.0), TMHMM v2.0 (https://services.healthtech.dtu.dk/service.php?TMHMM-2.0), and Big-GPI (https://mendel.imp.ac.at/gpi/fungi_server.html) software. Transcriptional factors were predicted through analyzing InterPro IDs in the Fungal Transcriptional Factors^63^. Default parameters were applied for all software tools.

### Identification of genes related to organic acid and polysaccharide biosynthesis

Genes encoding for proteins involved in organic acid and polysaccharide biosynthesis were predicted using the KEGG and BlastKOALA^19^. The questionable open reading frames were searched through BLASTp search (e-value cut-off = 1e−5) against the genome of strain C52 using reference protein sequences.

## Supplementary Information

Supplementary Information.

## Data Availability

The ITS gene sequence of strain C52 was deposited onto the GenBank (NCBI) under the accession number: MZ951162. The genome assembly of the *C. eragrostidis* C52 was deposited at the GenBank under the accession number number: JAIRCK000000000.
