# Supplementary material for: Genome-wide comparison deciphers lifestyle adaptation and glass biodeterioration property of Curvularia eragrostidis C52
Source: Sci Rep. 2022 Jul 6;12:11411. doi: 10.1038/s41598-022-15334-z (PMC9259613; doi:10.1038/s41598-022-15334-z)
Supplement: Supplementary file 1 — Supplementary Information. [file 41598_2022_15334_MOESM1_ESM.docx]

Supplementary Information

Genome-wide comparison deciphers lifestyle adaptation and glass biodeterioration property of *Curvularia eragrostidis* C52

# Ngoc Tung Quach1,2+, Cao Cuong Ngo1,3+, Thu Hoai Nguyen3, Phi Long Nguyen3, Thi Hanh Nguyen Vu1,2, Thi Hoai Trinh Phan4, Quang Huy Nguyen5, Thanh Thi Minh Le1, Hoang Ha Chu1,2 & Quyet-Tien Phi1,2,*

^1^Institute of Biotechnology, Vietnam Academy of Science and Technology, Hanoi 10000, Vietnam

^2^Graduate University of Science and Technology, Vietnam Academy of Science and Technology, Hanoi 10000, Vietnam

^3^Vietnam-Russia Tropical Centre, Hanoi 100000, Vietnam

^4^Department of Marine Biotechnology, Nhatrang Institute of Technology Research and Application, Vietnam Academy of Science and Technology, Nha Trang 650000, Vietnam

^5^LMI DRISA, Department of Life Sciences, University of Science and Technology of Hanoi, Vietnam Academy of Science and Technology, Hanoi 100000, Vietnam

*corresponding author: [tienpq@ibt.ac.vn](mailto:tienpq@ibt.ac.vn)

+these authors contributed equally to this work

**Tables and Figures**

**Table S1.** Biodeteriogenic potential of 12 fungal isolates from infected eyepieces of binoculars

| **Fungal isolate** | **MT1**  **(Initial pH 7.2)** | | **Hyphal surface**  **coverage (%)** | **EPS production (g/L)** | **Reduction of light transmission (%)** |
| --- | --- | --- | --- | --- | --- |
|  | **pH** | **Growth** |  |  |  |
| C11 | 2.71 | ++++ | 31.5±2.6 | 3.2±0.4 | 32.6±3.3 |
| C12 | 3.34 | +++ | 12.9±1.8 | 1.1±0.1 | 18.5±1.7 |
| C21 | 3.93 | +++ | 30.6±2.8 | 5.1±0.2 | 31.2±3.2 |
| C22 | 2.84 | +++ | 29.0±3.1 | 4.5±0.1 | 28.9±4.2 |
| C23 | 5.87 | +++ | 26.6±2.7 | 6.3±0.3 | 25.5±1.9 |
| C31 | 3.38 | ++++ | 31.4±3.5 | 13.1±0.8 | 41.3±2.3 |
| C32 | 3.49 | ++++ | 9.5±1.3 | 2.2±0.3 | 15.7±3.4 |
| C33 | 5.25 | +++ | 31.7±2.5 | 5.6±0.2 | 33.7±2.2 |
| C34 | 5.46 | +++ | 20.9±3.1 | 0.7±0.2 | 20.3±1.6 |
| C52 | 2.60 | ++++ | 46.2±3.3 | 19.0 ± 0.2 | 50.9±1.4 |
| C53 | 2.97 | +++ | 19.0±2.3 | 6.4±0.5 | 22.6±1.8 |
| C54 | 2.93 | ++++ | 16.7±2.1 | 8.7±0.4 | 24.2±1.9 |

Fair growth +; good growth ++; luxurious growth +++; heavy growth ++++; extreme growth +++++.

**Table S2.** EDS microanalysis of untreated and fungus-treated glasses

| **Element** | **Untreated glass** | | **Fungus-treated glass** | |
| --- | --- | --- | --- | --- |
|  | **Weight %** | **Atomic %** | **Weight %** | **Atomic %** |
| Silicon (Si) | 8.0±2.6 | 10.3±2.6 | 21.3±3.9 | 22.0±3.0 |
| Potassium (K) | 1.9±0.01 | 1.8±0.1 | 3.6±0.2 | 3.2±0.4 |
| Fluorine (F) | 22.2±1.1 | 42.5±5.4 | 15.8±0.3 | 20.3±6.1 |
| Magnesium (Mg) | 8.3±0.4 | 12.4±1.5 | 4.9±0.7 | 5.5±1.6 |
| Oxygen (O) | 10.1±3.5 | 22.7±6.3 | 22.4±3.0 | 41.3±4.5 |
| Sodium (Na) | **-** | **-** | 2.0±0.9 | 2.2±0.6 |
| Barium (Ba) | 3.8±0.8 | 1.0±0.3 | 3.9±3.0 | 1.6±0.4 |
| Hafnium (Hf) | 45.7±3.8 | 9.3±1.5 | 26.1±4.1 | 4.0±1.2 |

**Table S3.** Comparison of the main assembly and annotation features of *C. eragrostidis* C52 and other *Curvularia* genomes

| **Genome** | *C. eragrostidis* C52 | *C. geniculata* W-3 | *C. kusanoi* 30M1 | *C. lunata* CX-3 | *C. lunata* W3 | *C. papendorfii* UM 226 | *Curvularia* sp. IFB-Z10 | *C. geniculata* P1 |
| --- | --- | --- | --- | --- | --- | --- | --- | --- |
| **Isolation source** | Glass | Endophyte | Human | Endophyte | Endophyte | Human | Fish | Endophyte |
| **Sequencing**  **platform** | Illumina  MiSeq | Illumina HiSeq | Illumina HiSeq | Illumina HiSeq | Illumina HiSeq | Illumina  HiSeq | Illumina HiSeq | Illumina HiSeq |
| **Assembler** | SPAdes v3.15.1 | SPAdes  v. 3.9 | SPAdes  v. 3.13 | SOAPdenovo v.1.04 | SPAdes  v. 3.9 | SOAPdenovo v. 1.15 | Velvet  v. 1.27 | Unicycler  v. 0.4.8 |
| **Coverage** | 87X | 18X | 151X | 65X | 8X | 33X | 121X | 284X |
| **Assembly Accession** | GCA_020744315 | GCA_002982235 | GCA_011058905 | GCA_000743335 | GCA_005212705 | GCA_002161795 | GCA_000817285 | GCA_016162275 |
| **Contigs** | 3594 | 737 | 107 | 327 | 741 | 136 | 374 | 574 |
| **Largest contig** | 1,136,284 | 857,631 | 1,989,576 | 1,994,917 | 857,646 | 3,101,661 | 1,159,086 | 1,105,476 |
| **Total length** | 36,881,810 | 33,535,945 | 33,340,726 | 35,493,212 | 33,535,833 | 33,010,211 | 33,379,470 | 32,923,150 |
| **GC (%)** | 52.07 | 50.64 | 52.13 | 50.22 | 50.63 | 50.47 | 50.65 | 50.72 |
| **N50** | 230,720 | 226,947 | 1,018,367 | 788,415 | 226,891 | 1,949,676 | 146,099 | 247,944 |
| **N75** | 85,203 | 114,467 | 469,684 | 481,241 | 114,431 | 1,419,467 | 89,686 | 139,325 |
| **L50** | 52 | 44 | 12 | 16 | 48 | 7 | 68 | 40 |
| **L75** | 115 | 92 | 24 | 30 | 96 | 12 | 140 | 86 |

**Table S4.** Functional annotation and repetitive elements predicted in the *C. eragrostidis* C52 genome

| **Functional annotation (proteins)** | |
| --- | --- |
| GO | 8622 |
| COG | 14207 |
| Pfam | 10093 |
| InterPro | 11883 |
| KEGG | 7121 |
| **Interspersed repeat (sequences)** | 176 |
| SINEs | 20 |
| LINEs | 120 |
| LTR elements | 3 |
| DNA elements | 27 |
| **Tandem repeat** | 3959 |

**Table S5.** Comparison of COGs between *C. eragrostidis* C52 and other *Curvularia* strains

| **Class** | ***C. eragrostidis* C52** | ***C. geniculata* W-3** | ***C. kusanoi* 30M1** | ***C. lunata* CX-3** | ***C. lunata* W3** | ***C. papendorfii* UM 226** | ***Curvularia* sp. IFB-Z10** | ***C. geniculata* P1** |
| --- | --- | --- | --- | --- | --- | --- | --- | --- |
| (G) Carbohydrate transport and metabolism | 766 | 639 | 717 | 643 | 638 | 641 | 590 | 654 |
| (O) Posttranslational modification, protein turnover, chaperones | 647 | 616 | 653 | 581 | 620 | 595 | 588 | 624 |
| (S) Function unknown | 3262 | 2286 | 2371 | 2264 | 2306 | 2274 | 2073 | 2346 |
| (Q) Secondary metabolites biosynthesis, transport and catabolism | 713 | 556 | 556 | 556 | 562 | 540 | 480 | 566 |
| (K) Transcription | 517 | 397 | 404 | 391 | 407 | 416 | 368 | 409 |
| (J) Translation, ribosomal structure and biogenesis | 643 | 412 | 418 | 403 | 414 | 416 | 387 | 419 |
| (L) Replication, recombination and repair | 298 | 265 | 250 | 259 | 266 | 273 | 267 | 261 |
| (A) RNA processing and modification | 369 | 312 | 301 | 308 | 312 | 324 | 299 | 315 |
| (E) Amino acid transport and metabolism | 631 | 502 | 525 | 500 | 503 | 499 | 448 | 514 |
| (P) Inorganic ion transport and metabolism | 453 | 262 | 286 | 264 | 263 | 270 | 236 | 266 |
| (I) Lipid transport and metabolism | 497 | 376 | 381 | 370 | 384 | 389 | 347 | 385 |
| (B) Chromatin structure and dynamics | 93 | 113 | 114 | 107 | 113 | 120 | 109 | 115 |
| (T) Signal transduction mechanisms | 433 | 369 | 399 | 363 | 369 | 394 | 350 | 373 |
| (Z) Cytoskeleton | 309 | 150 | 139 | 157 | 155 | 158 | 156 | 150 |
| (U) Intracellular trafficking, secretion, and vesicular transport | 480 | 433 | 425 | 426 | 432 | 453 | 413 | 439 |
| (D) Cell cycle control, cell division, chromosome partitioning | 127 | 168 | 164 | 166 | 169 | 176 | 169 | 167 |
| (F) Nucleotide transport and metabolism | 190 | 126 | 128 | 120 | 128 | 127 | 111 | 125 |
| (M) Cell wall/membrane/envelope biogenesis | 118 | 86 | 94 | 93 | 88 | 100 | 91 | 94 |
| (C) Energy production and conversion | 585 | 391 | 398 | 374 | 394 | 378 | 336 | 395 |
| (H) Coenzyme transport and metabolism | 292 | 180 | 191 | 175 | 182 | 185 | 174 | 184 |
| (V) Defense mechanisms | 102 | 41 | 44 | 43 | 41 | 40 | 34 | 43 |
| (N) Cell motility | 4 | 7 | 5 | 6 | 7 | 7 | 8 | 7 |
| (Y) Nuclear structure | 47 | 27 | 27 | 25 | 27 | 29 | 26 | 27 |
| (W) Extracellular structures | 5 | 6 | 5 | 8 | 7 | 9 | 7 | 7 |

**Table S6.** List of predicted enzymes involved in organic acid production in the genome of

*C. eragrostidis* C52

| **Locus tag** | **Gene Name** | **EC number** | **Predicted function** |
| --- | --- | --- | --- |
| Orf_6854, Orf _8541 | *-* | 1.1.1.27 | Lactate/malate dehydrogenase |
| Orf _5034, Orf _8145, Orf _12974 | *mdh1* | 1.1.1.37 | Malate dehydrogenase |
| Orf _1459, Orf _5159 | *idH* | 1.1.1.41 | Isocitrate dehydrogenase NAD subunit, mitochondrial |
| Orf _10527 | *mrpL2* | 1.1.1.41 | Ribosomal L27 protein |
| Orf _2904 | *ipd1* | 1.1.1.42 | Isocitrate/isopropylmalate dehydrogenases |
| Orf _15056 | *icd* | 1.1.1.42 | Isocitrate dehydrogenase |
| Orf _5639 | *pda1* | 1.2.4.1 | Alpha subunit of pyruvate dehydrogenase |
| Orf _10050 | *pdb1* | 1.2.4.1 | Pyruvate dehydrogenase E1, beta subunit |
| Orf _11109 | *aceE* | 1.2.4.1 | Pyruvate dehydrogenase |
| Orf _6941 | *kgd1* | 1.2.4.2 | 2-oxoglutarate dehydrogenase |
| Orf _11904 | *odhA* | 1.2.4.2 | 2-oxoglutarate dehydrogenase, E1 component |
| Orf _13068 | *sucA* | 1.2.4.2 | 2-oxoglutarate dehydrogenase E1 component |
| Orf _11702 | *porG* | 1.2.7.3/1.2.7.11 | Pyruvate ferredoxin oxidoreductase gamma subunit |
| Orf _14759 | *MA20_35585* | 1.2.7.3 | Pyruvate ferredoxin/flavodoxin oxidoreductase |
| Orf _5130, Orf _9698 | *sdh12* | 1.3.5.1 | Succinate dehydrogenase complex, subunit B |
| Orf _10092 | *Sdh* | 1.3.5.1 | Succinate dehydrogenase |
| Orf _12446 | *sdhE* | 1.3.5.1 | Succinate dehydrogenase |
| Orf _12447 | *sdhB* | 1.3.5.1 | Succinate dehydrogenase/fumarate reductase |
| Orf _13412, Orf _14084, Orf _13412, Orf _14084 | *sdhA* | 1.3.5.1 | FAD-dependent oxidoreductase 2 family |
| Orf _11961 | *nadB* | 1.3.5.4 | L-aspartate oxidase |
| Orf _12446 | *sdhE* | 1.3.5.4 | Succinate dehydrogenase |
| Orf _12447 | *sdhB* | 1.3.5.4 | Succinate dehydrogenase/fumarate reductase |
| Orf _2311 | *lpd1* | 1.8.1.4 | Dihydrolipoamide dehydrogenase precursor |
| Orf _12208, Orf _14234 | *lpdA* | 1.8.1.4 | Dehydrogenase |
| Orf _12201, Orf _13503 | *lpd_1* | 1.8.1.4 | Belongs to the class-I pyridine nucleotide-disulfide oxidoreductase family |
| Orf _3223 | *pdx1* | 2.3.1.12 | Pyridoxine biosynthesis protein |
| Orf _4683 | *lat1* | 2.3.1.12 | Pyruvate dehydrogenase complex dihydrolipoamide acetyltransferase component (E2) |
| Orf _14071 | *aceF* | 2.3.1.12 | Pyruvate dehydrogenase E2 component |
| Orf _8555 | *kgd2* | 2.3.1.61 | 2-oxoacid dehydrogenases acyltransferase (catalytic domain) |
| Orf _14098 | *sucB* | 2.3.1.61 | 2-oxoglutarate dehydrogenase E2 component |
| Orf _3903, Orf _9295, Orf _11022, Orf _14255 | *cit* | 2.3.3.1 | Citrate synthase |
| Orf _12445 | *gltA* | 2.3.3.1 | Citrate synthase |
| Orf _12690 | *prpC* | 2.3.3.1 | Citrate synthase |
| Orf _1747, Orf _1748 | *acl2* | 2.3.3.8 | Citrate synthase, C-terminal domain |
| Orf _13082 | *pckG* | 4.1.1.32/ 4.1.1.49 | Phosphoenolpyruvate carboxykinase |
| Orf _4338 | *pck1* | 4.1.1.49 | Phosphoenolpyruvate carboxykinase |
| Orf _1108 | *fum1* | 4.2.1.2 | Fumarase C, C-terminus |
| Orf _11038 | *fumA* | 4.2.1.2 | Fumarase A |
| Orf _11683 | *fumC* | 4.2.1.2 | Fumarate hydratase class II |
| Orf _5714 | *aco* | 4.2.1.3 | Aconitase |
| Orf _11512, Orf _12715 | *acn* | 4.2.1.3 | Aconitate hydratase |
| Orf _2583 | *-* | 6.2.1.4 | CoA binding domain |
| Orf _2848, Orf _2849, Orf _8817 | *lsc1* | 6.2.1.4 | Succinyl-CoA synthetase alpha subunit |
| Orf _8817 | *lsc2* | 6.2.1.4 | Succinyl-CoA synthetase beta subunit |
| Orf _2559 | *-* | 6.2.1.5 | CoA binding domain |
| Orf _12185, Orf _12932, Orf _14576, Orf _14643 | *sucD* | 6.2.1.5 | Succinyl-CoA ligase subunit alpha |
| Orf _12186 | *sucC* | 6.2.1.5 | Succinyl-CoA synthetase beta subunit |
| Orf _5204, Orf _5205,  Orf _11122, Orf _12135 Orf_12853 | *pyc/mccA* | 6.4.1.1 | Pyruvate carboxylase |


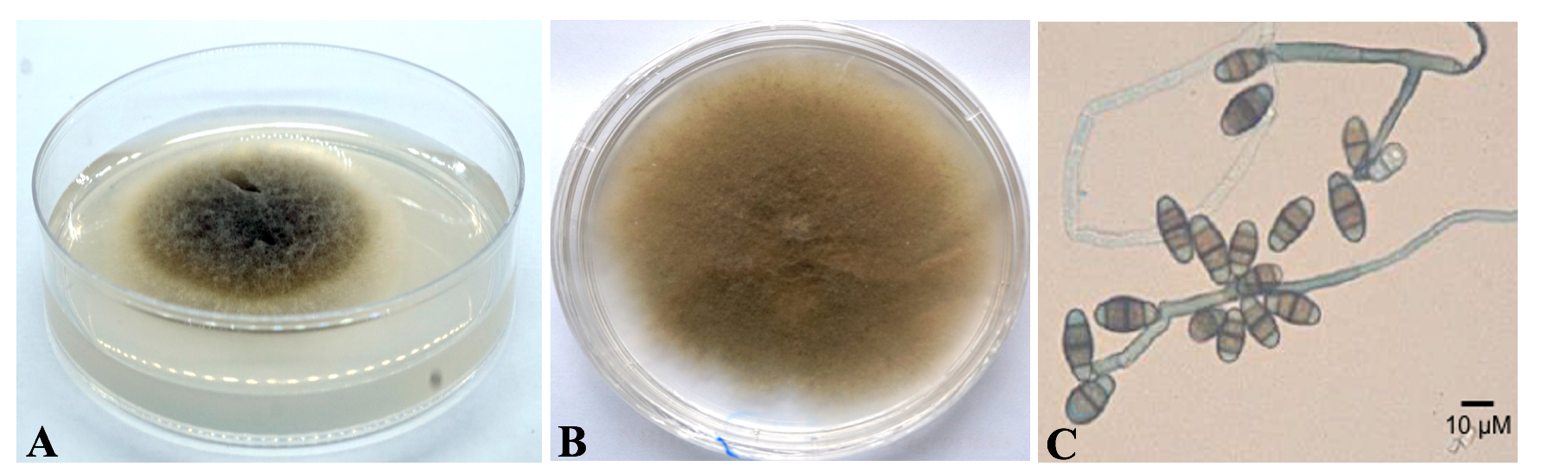


**Fig. S1.** Colony morphology observed on Czapek–Dox agar after 5 days **(A)** and 12 days **(B)**. Scanning electron micrograph conidia formed on the conidiophores **(B).**


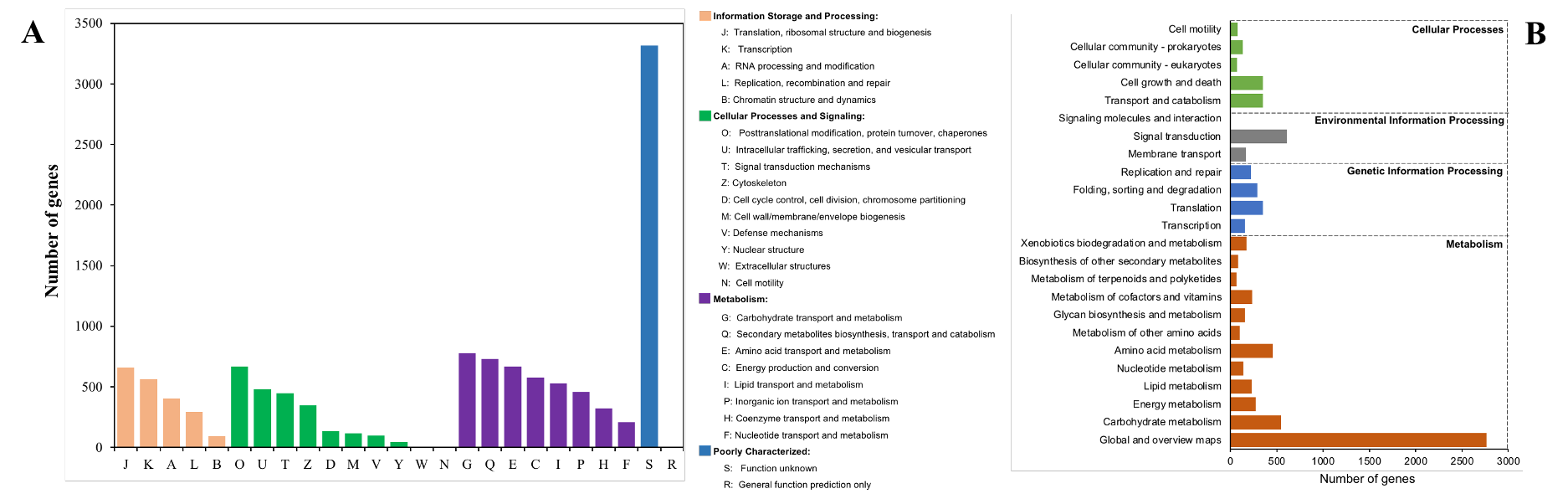


**Fig. S2.** Clusters of Orthologous Groups (COG) **(A)** and Kyoto Encyclopedia of Genes and Genomes (KEGG) **(B)** functional annotation of *C. eragrostidis* C52.


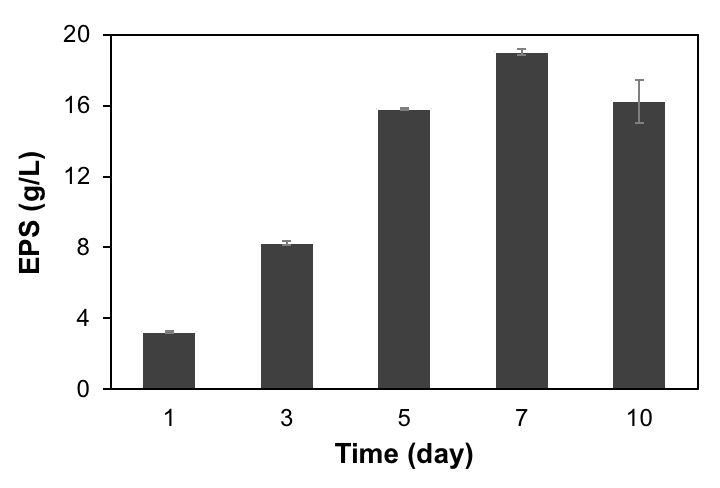


**Fig. S3.** EPS production observed in *C. eragrostidis* C52 at different incubation times.


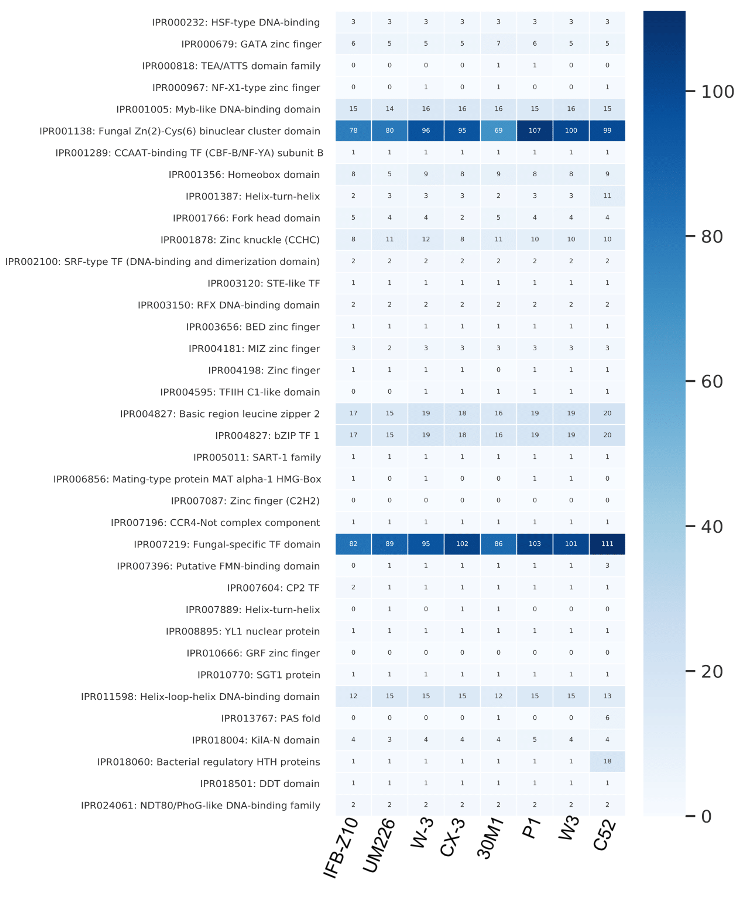


**Fig. S4.** The number of transcription factors identified in all *Curvularia* genomes: C52, *C. eragrostidis* C52; W-3, *C. geniculata* W-3; 30M1, *C. kusanoi* 30M1; CX-3, *C. lunata* CX-3; W3, *C. lunata* W3; UM226, *C. papendorfii* UM 226; P1, *C. geniculata* P1; IFB-Z10, *Curvularia* sp. IFB-Z10.
